# Longitudinal Study of Delta-Aminolevulinate Dehydratase Activity and Oxidative Profile in Healthy Pregnant Women

**DOI:** 10.3390/biom9010018

**Published:** 2019-01-09

**Authors:** Leidiane de Lucca, Letícia Bigolin Jantsch, Silmara Ana Vendrame, Carolina dos Santos Stein, Vanessa Cristina Grólli Klein, Karina Biaggio Soares, Francisco Maximiliano Pancich Gallarreta, Rafael Noal Moresco, Thissiane de Lima Gonçalves

**Affiliations:** 1 Postgraduate Program in Pharmaceutical Sciences, Department of Clinical and Toxicology Analysis, Center of Healthy Sciences, Federal University of Santa Maria (UFSM), Santa Maria 97105-900, Brazil; leidi_lucca@hotmail.com (L.d.L.); letii_jantsch@hotmail.com (L.B.J.); silmaravendrame@yahoo.com.br (S.A.V.); stein.carolina@gmail.com (C.d.S.S.); rnmoresco@ufsm.br (R.N.M.); 2 Department of Obstetrics and Gynecology, Federal University of Santa Maria (UFSM), Santa Maria 97105-900, Brazil; nessakleinrs@yahoo.com.br (V.C.G.K.); karinabiaggiosoares@icloud.com (K.B.S.); fmgallarreta@msn.com (F.M.P.G.)

**Keywords:** antioxidants, free radicals, oxidative stress, pregnant women, δ-ALA-D

## Abstract

Pregnancy is characterized by changes in various organs, triggering changes in the use of energy substrates and increased oxygen consumption. In addition, gestation is an oxidative event that can be assessed by the relationship between free radicals and antioxidants produced by the body. Excessive production of free radicals has detrimental effects such as damage to enzymes, carbohydrates, and DNA. Thus, the objective of this study was to evaluate the oxidative status and antioxidant responses throughout pregnancy through a longitudinal study. Reactive oxygen species were analyzed by means of thiobarbituric acid reactive substances and nitric oxide, the antioxidant system through vitamin C, sulfhydryl groups, total antioxidant capacity, and ferric reducing ability of plasma as well as enzymes such as catalase and delta-aminolevulinate-dehydratase in pregnant women in the three gestational trimesters (*n* = 30). According to the results, the markers of oxidative damage showed significant differences in the different gestational trimesters where they were increased in the second trimester when compared to the first trimester. The antioxidant defenses responded differently in each gestational trimester, suggesting a response pattern to try to combat the damage caused by free radicals, in order to stabilize the increase of oxidative stress caused in the second gestational trimester.

## 1. Introduction

Gestation is characterized by a series of transformations associated with anatomical, physiological, and metabolic adaptations. These changes are necessary since they favor the supply of metabolites and nutrients to the fetus [1]. These various changes lead to changes in the use of energetic substrates and a greater consumption of oxygen [2]. Due to this, a state of high degree oxidative stress occurs [3], and this state is regulated by several physiological mechanisms in order to maintain a continuous balance between the production of free radicals and the antioxidant capacity of the pregnant woman in order to allow the normal progression of gestation and fetal growth [4]. Due to this, it is very important to monitor these changes in all pregnant women to improve the quality of life of the mother and the newborn [1].

The placenta is the main source of pro-oxidants because it presents intense cellular activity as it is an environment rich in mitochondria, highly vascularized [5,6], and dependent on the availability of oxygen, favoring the development of oxidative stress [6,7]. In addition, oxidative stress can reach extreme levels during placental development and may be related to several pathologies in pregnancy [8], playing a role in restricting intrauterine growth [9], fetal death, spontaneous abortion and preeclampsia, among others [10,11].

Oxidative stress is characterized by a loss of balance between pro-oxidants and antioxidants. This imbalance can occur due to a number of factors such as the over-production of free radicals, overloading the amount of antioxidants available, or an insufficient amount of circulating antioxidants, leading to low body defense against the harmful effects of free radicals [11,12,13]. Due to its great ability to irreversibly oxidize some cellular components such as deoxyribonucleic acid (DNA), carbohydrates, lipids, and proteins, oxidative stress is considered to be one of the most frequent mechanisms associated with the pathophysiology of some diseases as well as some physiological conditions such as gestation [10,14,15,16,17].

Enzymes such as catalase and superoxide dismutase along with dietary antioxidants like vitamin C and α-tocopherol constitute the body’s primary antioxidant defense against oxidative damage [10]. In addition, thiol proteins actively participate in redox regulation, being achieved by a combination of cysteine oxidation by hydrogen peroxide or other reactive oxygen species, and the main reducing systems are represented by thioredoxins, glutaredoxins, and peroxiredoxins [18,19]. The sulfhydryl delta-aminolevulinate dehydratase (δ-ALA-D) enzyme that forms part of the heme biosynthetic route in pro-oxidant situations may be inhibited such as preeclampsia and gestational diabetes [10,17,20,21]. This is due to the fact that this enzyme is responsible for catalyzing the asymmetric condensation of two molecules of 5-aminolevulinic acid (ALA), resulting in the formation of the monopyrrole porphobilinogen (PBG). In the following steps, PBG is mounted on tetrapyrrole molecules, which integrate the prosthetic groups of some proteins like hemoglobin, cytochromes, and enzymes like catalase. In situations where there is an increase in oxidative stress, the activity of the enzyme δ-ALA-D may be reduced and this may lead to enzyme substrate accumulation (ALA), which, in turn, may exacerbate the production of reactive oxygen species (ROS), contributing to oxidative stress. Due to this, this enzyme may be suggested as an indirect marker of oxidative stress and impairment of metabolic processes because the modulation of the activity of this enzyme contributes significantly to the global level of oxidative stress [15,16,20].

There is evidence that shows changes in oxidative stress during healthy pregnancy and in pregnant women with obstetric complications. The interest in these studies has intensified in recent years due to the possible involvement of oxidative and antioxidant substances in the physiology of various processes and some pathologies. However, to date, there have been scarce indications in the literature about these changes in the three trimesters of gestation and previous studies have reported conflicting results. Therefore, the objective of this study was to evaluate the oxidative, biochemical, and hematological profile in pregnant women without obstetric complications through a longitudinal study.

## 2. Materials and Methods

### 2.1. Study Population

Pregnant women attending the basic health units of the municipality of Santa Maria, Rio Grande do Sul, Brazil were recruited and invited to the study by doctors responsible for prenatal care from the authorization of the person in charge of the unit as well as the Nucleus of Permanent Education in Health (NEPeS), and the municipal health secretariat. A total of 57 pregnant women agreed to participate in the research. After acceptance, the free and informed consent form was read and signed by all participants. This longitudinal study was approved by the Department of Education, Research, and Extension (DEPE) of the University Hospital of Santa Maria, later by the Research Ethics Committee (CEP) of the Federal University of Santa Maria (UFSM), and received the Presentation Certificate for Assessment Ethics under number: 62643616.2.0000.5346.

**Figure 1:**
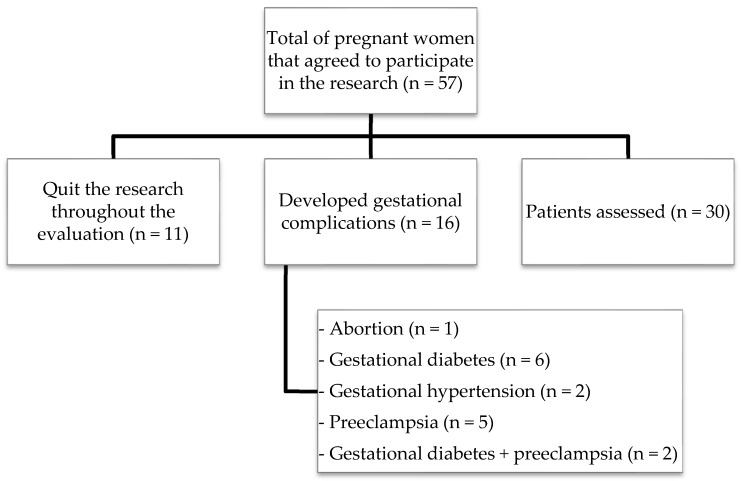
Flowchart of patients included in the study.

The pregnant women were recruited in the first trimester and followed up until the end of gestation, between February 2017 and June 2018. The inclusion criteria were: (1) gestational age less than 14 weeks; (2) maternal age between 18 and 40 years; and (3) single gestation (Figure 1). Women with preexisting chronic diseases, smokers, alcoholics, and participants that developed some gestational complications such as abortion, gestational diabetes, gestational hypertension, and/or preeclampsia were excluded from the present study (Figure 1).

### 2.2. Sample Collection

Blood and urine were collected from the study participants on three separate occasions during pregnancy: in the first trimester (13 (12–14) weeks of gestation), second trimester (22 (21–23) weeks of gestation), and in the last gestational trimester (30 (30–31) weeks of gestation). Samples were collected after 8 h of fasting. Blood was collected in vacuum tubes containing EDTA, sodium fluoride, and heparin or without anticoagulant. The whole blood, plasma, and erythrocytes used in the determinations were obtained from tubes containing heparin, the plasma was separated by centrifugation at 3000 rpm, and the erythrocytes were washed with 0.9% sodium chloride. Plasma, serum, and urine aliquots were stored at −80 °C for subsequent evaluation of nitric oxide (NOx), total antioxidant capacity (TAC), ferric reducing ability of plasma (FRAP), urinary creatinine, urinary albumin, and urinary protein, and other tests were processed immediately after collection.

### 2.3. Clinical and Hematological Profile

Gestational age was determined according to the data obtained on the first ultrasound. Blood pressure was evaluated in an aneroid sphygmomanometer and body mass index (BMI) was calculated by dividing weight by squared height (kg/m^2^). Hematological parameters (erythrocytes, hemoglobin, hematocrit, leukocytes, and platelets) were evaluated in blood collected with EDTA and determined with an automatic cell counter Sysmex XE 5000^®®^ (Sysmex Corporation, Kobe, Japan).

### 2.4. Biochemical Profile

Glucose was determined on a fasting blood sample, measured in plasma obtained from a sodium fluoride tube, and evaluated by the standard method with a commercial kit (Bioclin^®®^). Urinary creatinine, urinary albumin, and urinary protein levels were measured using the Bioclin^®®^ kit. Urinary creatinine was measured through a kinetic method, urinary albumin levels were measured by an immunoturbidimetric method, and urinary protein levels using a colorimetric method.

### 2.5. Oxidative Profile

Lipid peroxidation was evaluated in the plasma and erythrocytes by quantification of thiobarbituric acid reactive substances (TBARS) according to the method of Lapenna et al. [22], and the results expressed in nmol MDA/mL plasma and nmol MDA/mL of erythrocytes. The dosage of nitric oxide (NOx), based on the accumulation of nitrite/nitrate in the serum, was measured according to the methodology of Tatsch et al. [23] and the results presented in μmol/L.

Protein thiol groups (P-SH in plasma) and non-protein thiol groups (NP-SH in erythrocytes) were evaluated according to Boyne and Ellman [24] as modified by Jacques-Silva et al. [25], and the results expressed as nmol P-SH/mL plasma and nmol NP-SH/mL erythrocytes. Vitamin C in plasma was determined as described by Galley et al. [26] with some modifications by Jacques-Silva et al. [25] and the results presented in μg vit C/mL plasma. The activity of the enzyme catalase was measured spectrophotometrically by the method of Aebi et al. [27] and the results expressed as K/mg Hb.

The evaluation of the ferric reducing ability of plasma (FRAP) was determined according to Benzie and Strain [28] and the results presented in μmol/L. The evaluation of the total antioxidant capacity (TAC) in the serum was performed according to the method of Erel [29] with modifications and results expressed in mmol Trolox Equivalent/L.

The δ-ALA-D activity was determined in whole blood by the method of Berlin and Schaller [30] and the results are expressed as U/L (nmol PBG/h/mg Hb). A similar set of tubes was incubated with the addition of 2 mM dithiothreitol (DTT), which is a reducing agent, to verify if the changes in the enzymatic activity were related to the oxidation of thiolic groups and to obtain the reactivation index using A-B/A × 100, where A = DTT absorbance assay and B = no DTT absorbance assay.

### 2.6. Statistical Analysis

The clinical, hematological, biochemical, and oxidative profiles of the participants were statistically analyzed using Graph Pad Prism v.6 software. The Shapiro-Wilk test was used to verify the distribution of the sample. When groups were distributed normally, we used one-way analysis of variance (ANOVA) followed by Tukey’s test for comparison between groups and the data were presented as mean ± standard deviation (SD). For analysis of non-parametric data, the Kruskal-Wallis test was used and the results expressed as median (interquartile range). Values of *p* < 0.05 were considered statistically significant for all analyses.

## 3. Results

A total of 30 pregnant women who did not present any gestational complications participated in all stages of the research. The clinical and demographic parameters evaluated in the participants are shown in Table 1. As already expected, the weights and BMIs of the pregnant women increased throughout the gestation. The other parameters evaluated in the present study did not show significant differences during the gestational trimesters.

**Figure 2:**
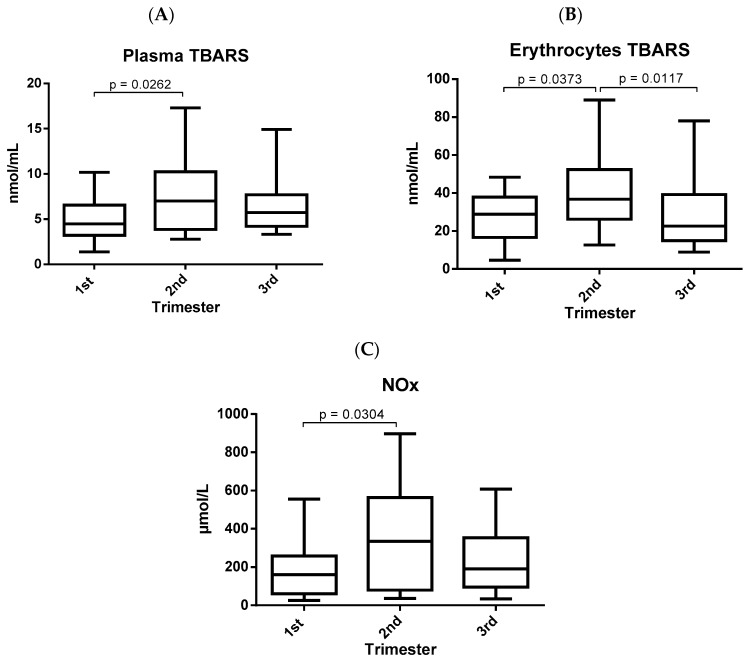
Markers of oxidative damage in the three gestational trimesters. Data are presented as median with interquartile range. Statistically significant differences were determined by Kruskal-Wallis. (**A**) TBARS levels in plasma; (**B**) TBARS levels in erythrocytes; (**C**) dosage of nitric oxide; TBARS: thiobarbituric acid-reactive substances; NOx: nitric oxide.

The markers of oxidative damage assessed in pregnant women presented significant differences in the different gestational trimesters and are shown in Figure 2. The three markers were increased in the second trimester when compared to the first trimester. However, only erythrocyte TBARS (Figure 2B) decreased significantly in the third trimester when compared to the second gestational trimester.

**Figure 3:**
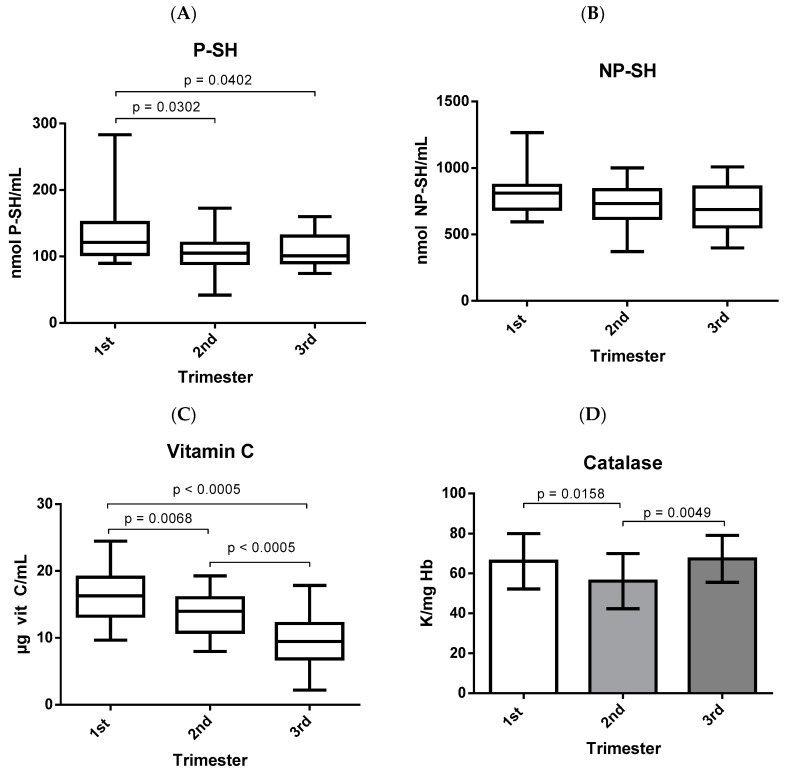

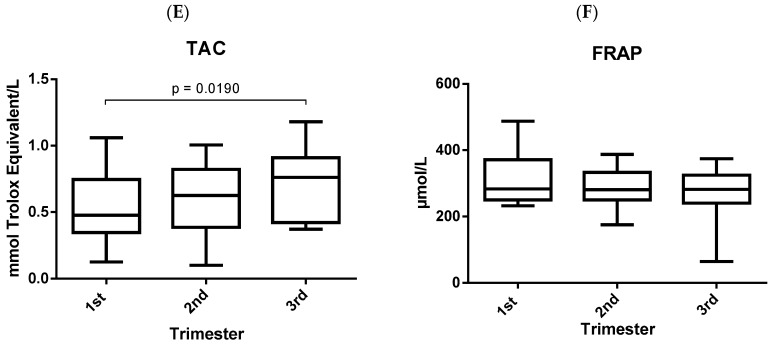
Antioxidant defenses in the three gestational trimesters. P-SH, NP-SH, vitamin C, TAC, and FRAP were analyzed by Kruskal-Wallis and represented as median with interquartile range. Catalase was analyzed by ANOVA followed by Tukey’s test and represented as mean with standard deviation. (**A**) thiol groups in plasma; (**B**) non-protein thiol groups in erythrocytes; (**C**) vitamin C in plasma; (**D**) activity of the enzyme catalase; (**E**) evaluation of the total antioxidant capacity; (**F**) evaluation of ferric reducing ability of plasma; FRAP: ferric reducing ability of plasma; P-SH: protein thiol groups; NP-SH: non-protein thiol groups; TAC: total antioxidant capacity.

Antioxidant defenses responded differently in each gestational trimester and are shown in Figure 3. Plasma thiolic groups were decreased in the second and third trimesters when compared to the first trimester of gestation (Figure 3A). However, the clusters of thiols in the erythrocytes did not show significant differences during pregnancy (Figure 3B). Vitamin C decreased during the three gestational trimesters (Figure 3C), showing significant differences in the second and third trimesters when compared to the first trimester and, in addition, there was a significant difference between the third and second gestational trimesters. On the other hand, the activity of the enzyme catalase decreased significantly in the second trimester when compared to the first trimester; however, in the third trimester, there was an increase in enzyme activity, which was significantly higher when compared to the second trimester (Figure 3D). However, the total antioxidant capacity increased throughout the gestational trimesters, presenting a significant increase in the third trimester when compared to the first trimester of gestation (Figure 3E). However, the FRAP remained stable throughout the three gestational trimesters, with no significant differences between the quarters (Figure 3F).

**Figure 4:**
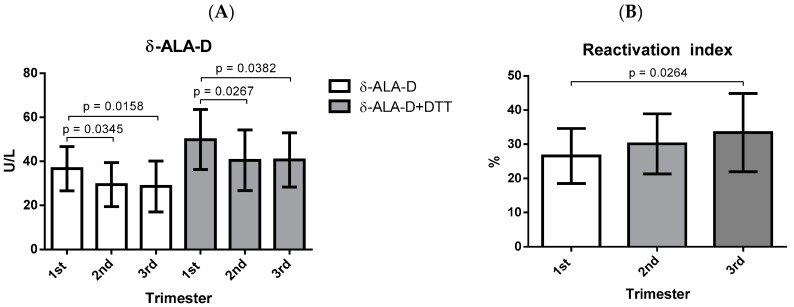
δ-ALA-D activity and reactivation index of the δ-ALA-D enzyme in the three gestational trimesters. The data were determined by ANOVA followed by Tukey’s test and represented as mean with standard deviation. (**A**) δ-ALA-D activity; (**B**) reactivation index of δ-ALA-D enzyme; δ-ALA-D: delta-aminolevulinate dehydratase enzyme; DTT: dithiothreitol.

With regard to the activity of the δ-ALA-D enzyme, there was a decrease in the second and third trimesters of gestation when compared to the first trimester, as can be observed in Figure 4A, which was also observed after the addition of DTT (Figure 4A). However, the rate of enzymatic reactivation was significantly higher in the third trimester when compared to the first gestational trimester (Figure 4B).

## 4. Discussion

One of the most frequent physiological alterations during gestation is an increase in oxidative stress when compared to not pregnant [8,10,31]. Bukhari et al. [31] reported significant differences in the parameters of oxidative stress since the first gestational trimester. In addition, several studies have attributed an important participation in gestational complications to the exaggerated oxidative damage such as repetitive abortion, prematurity, preeclampsia, and gestational diabetes, among others [8,10,13,32]. Few studies have evaluated the longitudinal change in oxidative stress and the antioxidant system during the three trimesters of gestation, however, the results found in these studies present discordant data. According to the results obtained in the present study where differences in the δ-ALA-D activity were demonstrated for the first time, it is assumed that there is a different oxidative response profile in each gestational trimester with alterations in oxidative and antioxidant markers.

The study of mechanisms for regulation and formation of free radicals in the body is very relevant to understand the cellular changes that are directly linked to the control of proliferation, survival, and cell death [10,12]. The production of these radicals can occur in situations of inflammation-infection, hypoxia-hyperoxia, and ischemia-reperfusion as well as physiologically, through the cellular metabolism of acids and prostaglandins [33]. The free radicals produced are highly reactive and unstable molecules, they have a great capacity to interact with biomolecules (lipids, proteins, carbohydrates, and nucleic acids) in order to acquire molecular stability. Thus, these biomolecules end up undergoing oxidation, generating cellular damages such as changes in membrane, DNA, endoplasmic reticulum, protein denaturation and lipid peroxidation, further favoring the production of reactive oxygen species [12,34,35].

The biochemical events caused by the action of ROS on the unsaturated lipids of cell membranes can be characterized by lipid peroxidation. One of the main markers of oxidative stress for assessing lipid damage is malondialdehyde (MDA), which is formed through the oxidation of polyunsaturated fatty acids, mainly arachidonic acid [13,17,36,37]. Previous studies have reported conflicting results pertaining to MDA levels in pregnancy, Yüksel et al. [38] verified a decrease in MDA levels in the third trimester, whereas in contrast, Bukhari et al. [31] demonstrated an increase through pregnancy. The produced MDA can be quantified through the TBARS technique as it reacts easily with thiobarbituric acid (TBA) [37,39]. In spite of that, in the present study, increased TBARS levels in plasma (Figure 2A) and erythrocytes (Figure 2B) were observed in the second trimester when compared to the other two gestational trimesters. MDA is a highly toxic product and easily interacts with biomolecules and can even cause cell death, so if it is not eliminated it can lead to pathological processes [39,40].

Nitric oxide (NO) is one of the most important mediators of the endothelial system due to its antiplatelet, vasodilator, anti-proliferative, anti-inflammatory, and permeability reduction abilities. It acts as a free radical and its synthesis is performed by enzymatic action through nitric oxide synthase (NOs) from l-arginine, which produces l-citrulline and nitric oxide, being dependent on two cofactors: oxygen and phosphate dinucleotide adenine nicotinamide (NADPH) [41,42]. In addition, NO can be produced via the reduction of nitrite under hypoxia conditions to regulate a spectrum of physiological responses in the blood and tissues [43]. Yüksel et al. [38] reported an increase in NO levels in the third trimester. However, the level of nitric oxide in the serum of pregnant women, indicated by the concentration of nitrite/nitrate, evaluated in this study, was increased in the second trimester of pregnancy when compared to the first trimester (Figure 2C). NO involved in the vasodilation and vascular system [41,42] may be a justification for the NO levels being elevated in the second trimester given the presence of a highly vascularized placenta. High nitric oxide concentrations can indirectly cause deleterious effects through interactions with the superoxide radical, thus generating a highly reactive and potent nitrogen species called peroxynitrite [44].

To reduce or reverse the damage caused by free radicals, the body has antioxidant systems that can act with various mechanisms of action to prevent the formation of these species, inhibit the action of radicals, or repair the damage caused by biomolecules. The antioxidant system can be classified as enzymatic or non-enzymatic, acting in several ways [3,45]. Examples of non-enzymatic antioxidants are the thiol compounds, which are antioxidant substances possessing the thiol group (-SH) within their structure such as cysteine, glutathione, and thiol proteins [46,47]. As observed in Figure 3A, thiol groups in plasma were decreased in the second and third trimesters when compared to the first gestational trimester. These compounds are involved in the various physiological functions and mechanisms of some pathologies due to their ability to chelate harmful metal ions and sequester free radicals, playing a crucial role in protecting against oxidative damage in plasma and erythrocytes [46,47]. However, according to the results obtained in the present study, vitamin C decreased during gestation, showing lower concentrations in the third gestational trimester (Figure 3C). This could be justified as the vitamin is a reducing agent, and is considered as a “preventative” or “primary” antioxidant because it acts as a first-line defense mechanism for the plasma and cytosol. It reacts with free radicals such as superoxide and hydrogen peroxide, by donating electrons to destroy them before they react with lipoproteins and membranes, avoiding lipid peroxidation and the beginning of the oxidative process [48,49].

Catalase is an enzyme that is present in most aerobic cells, expressed mainly in peroxisomes, and exerts its activity on erythrocytes, liver, kidneys, and adipose tissue by acting to decompose H_2_O_2_, in addition to preventing the accumulation of methemoglobin [35,50,51]. As observed in Figure 3D, catalase activity was significantly lower in the second trimester when compared to the first trimester, confirming the results obtained by Yüksel et al. [38]. However, enzyme activity increased significantly in the third trimester when compared to the second gestational trimester, which is relevant for gestation since the normal activity of this enzyme is of extreme importance due to the fact that excess H_2_O_2_ causes an increase in oxidative stress as well as the oxidation of hemoglobin, leading to a decrease in oxygen concentration which can lead to infections, the formation of ulcers, and even necrosis [52].

Determining the total antioxidant capacity has the main advantage of measuring the antioxidant activity of all of the biological components, whether endogenous or exogenous, and not only of an isolated compound. In addition, it can add information about species that are unknown or difficult to measure [29,53]. This can justify the difference in the behavior of TAC observed in the present study when compared to the other antioxidant parameters, because according to the results obtained here, the TAC is higher in the third trimester when compared to the first gestational trimester (Figure 3E), confirming the results obtained by Bukhari et al. [31]. In addition, this increase in TAC throughout pregnancy may be related to the decrease in TBARS and NOx levels in the third trimester, where the TAC may be acting to reverse the increase in oxidative stress observed in the second gestational trimester. However, the FRAP remained stable during gestation (Figure 3F), and this parameter evaluates the reduction capacity of ferric ions by the action of the sample with the potential for reducing activity [53].

The activity of δ-ALA-D is strongly related to oxidative stress, since it is very sensitive to pro-oxidant elements, which act on the thiol groups of the enzyme resulting in decreased activity and, consequently, affecting heme synthesis [20]. In our study, pregnant women presented lower δ-ALA-D activity in the second and third trimesters when compared to the first gestational trimester (Figure 4A), which may contribute to other alterations observed in the different parameters of oxidative stress evaluated in the study. One such instance is that the levels of ALA may be elevated and this acid undergoes auto-oxidation at physiological pH, favoring the formation of free radicals such as H_2_O_2_, O_2_, and ALA, which may cause oxidative damage to DNA, increase lipid peroxidation, and decrease the antioxidant system [16,20]. In addition, after incubation with DTT, which is a reducing agent, it was possible to observe changes in enzyme activity (Figure 4A), suggesting that DTT was able to prevent the oxidation of δ-ALA-D sulfhydryl groups [54] since this compound is commonly used to maintain the thiol groups of the enzyme in a reduced state, reversing the inhibition of the δ-ALA-D activity and, thus, is able to verify the rate of enzymatic reactivation [16,20]. As shown in Figure 4B, the reactivation rate was higher in the last gestational trimester when compared to the first trimester, suggesting a possible involvement of the thiol groups in the restructuring of the enzyme activity after the addition of DTT in vitro.

During pregnancy, there is a state of high oxidative stress [8], which is regulated by several physiological mechanisms in order to maintain a continuous balance between the production of free radicals and the antioxidant capacity of the pregnant woman to allow the normal progression of gestation and fetal growth [4]. This may justify the different response patterns observed in the antioxidants evaluated in the present study. The placenta is the main source of pro-oxidant agents due to intense cellular activity, has numerous mitochondria, is highly vascularized [5], and is dependent on the availability of oxygen, favoring the development of oxidative stress [7]. In addition, the placenta is primarily responsible for providing nutrients and oxygen to the fetus and placentation begins between the 8^th^ and 18^th^ week of gestation. In a normal pregnancy, it is at this stage that a layer of trophoblastic cells invades the uterine wall in order to modify the structure of the spiral arteries of the pregnant woman. The muscular layer of the spiral arteries is replaced by a fibroid layer through the trophoblastic cells, with a decrease in vascular resistance and an increase in vessel diameter, thus allowing adequate blood perfusion to the fetus [55]. Thus, the trophoblastic invasion changes the uterine hemodynamics from a low flow system with high resistance to a high flow and low resistance system, causing a physiological increase in oxidative stress [8,56], which may justify the increase in oxidative stress observed mainly in the second gestational trimester and then the reduction of oxidative stress in the third trimester through the decrease in erythrocyte TBARS and increase in the catalase activity. This suggests a possible recovery of the organism against oxidative damage in the third trimester, in search of oxidative balance until the end of gestation.

## 5. Conclusions

The present study suggests that oxidative stress levels respond differently in each gestational trimester, where markers of oxidative damage appear increased in the second gestational trimester. On the other hand, antioxidant defenses respond in different ways in the different trimesters of gestation, and these substances act together with the main objective to revert and/or prevent the possible damages caused by the increase of oxidative stress occurring in the second trimester. In addition, this study highlights the activity of δ-ALA-D and its possible relationship with changes observed in other markers of oxidative stress in pregnant women. As oxidative stress and changes in antioxidant capacity may be related to several gestational complications, these results are relevant for a better understanding of the physiology of gestation.

## Figures and Tables

**Table 1:** Clinical and demographic characteristics in pregnant women in the three gestational trimesters.

Parameter	1st Trimester (*n* = 30)	2nd Trimester (*n* = 30)	3rd Trimester (*n* = 30)
Age (years)	26.72 ± 4.18	-	-
Gestational age (weeks)	13 (12–14)	22 (21–23)	30 (30–31)
Weight (kg)	63.77 ± 10.47	67.49 ± 10.07	71.54 ± 10.63 *
BMI (kg/m^2^)	23.94 ± 3.79	25.36 ± 3.78	26.89 ± 4.02 *
Systolic pressure (mmHg)	110 (100–120)	110 (100–110)	110 (100–110)
Diastolic pressure (mmHg)	60 (60–70)	60 (60–70)	60 (60–70)
Erythrocytes (10^6^/mm^3^)	4.19 ± 0.32	4.07 ± 0.33	3.99 ± 0.39
Hemoglobin (g/dL)	12.39 ± 0.93	12.12 ± 0.95	11.94 ± 1.06
Hematocrit (%)	36.69 ± 2.94	35.83 ± 2.93	35.29 ± 3.14
Platelets (10^3^/mm^3^)	235.9 ± 46.6	225.9 ± 47.8	219.7 ± 49.1
Leukocytes (/mm^3^)	8999 ± 1991	9307 ± 1889	9852 ± 2922
Glucose (mg/dL)	82.60 ± 7.70	80.80 ± 6.46	79.23 ± 7.26
Urinary creatinine (mg/dL)	92.75 (44.44–131.20)	70.38 (41.63–99.13)	70.50 (46.50–100.80)
Urinary albumin (mg/L)	4.55 (2.79–14.20)	4.715 (1.99–7.68)	7.72 (3.58–11.39)
Urinary protein (mg/L)	4.35 (1.17–5.65)	3.40 (1.42–5.50)	5.50 (2.57–6.53)

Parametric results were determined by ANOVA followed by Tukey’s test and represented as mean ± standard deviation and nonparametric results were determined by the Kruskal-Wallis test and represented as median (interquartile range). * *p* < 0.05 when compared to the 1st trimester. BMI: Body Mass Index.
